# The Impact of Limited Prosthetic Socket Documentation: A Researcher Perspective

**DOI:** 10.3389/fresc.2022.853414

**Published:** 2022-03-07

**Authors:** Jennifer Olsen, Shruti Turner, Alix Chadwell, Alex Dickinson, Chantel Ostler, Lucy Armitage, Alison H. McGregor, Sigrid Dupan, Sarah Day

**Affiliations:** ^1^Intelligent Sensing Laboratory, School of Engineering, Newcastle University, Newcastle upon Tyne, United Kingdom; ^2^Sackler MSk Laboratory, Department of Surgery and Cancer, Sir Michael Uren Hub, Imperial College London, London, United Kingdom; ^3^Human Movement and Rehabilitation Research Group, University of Salford, Salford, United Kingdom; ^4^Faculty of Engineering and Physical Science, University of Southampton, Southampton, United Kingdom; ^5^Portsmouth Enablement Centre, Portsmouth Hospital University National Health Service (NHS) Trust, St Marys Hospital, Portsmouth, United Kingdom; ^6^School of Mechanical, Materials, Mechatronic and Biomedical Engineering, University of Wollongong, Wollongong, NSW, Australia; ^7^Edinburgh Neuroprosthetics Laboratory, School of Informatics, University of Edinburgh, Edinburgh, United Kingdom; ^8^Department of Biomedical Engineering, University of Strathclyde, Glasgow, United Kingdom

**Keywords:** prosthetics, sockets, documentation, socket fit, prosthetic socket

## Abstract

The majority of limb prostheses are socket mounted. For these devices, the socket is essential for adequate prosthetic suspension, comfort, and control. The socket is unique among prosthetic components as it is not usually mass-produced and must instead be custom-made for individual residual limbs by a prosthetist. The knowledge of what constitutes “good” socket fit is gained by expert prosthetists and technicians over years of experience, and rarely documented. The reliance on tacit knowledge makes it difficult to standardize the criteria for a well-fitting socket, leading to difficulties understanding the impact of socket fit. Despite its importance, the workflow for socket fitting is often overlooked in literature. Due to the customized nature of sockets, if information is provided in literature, generally only the type of socket and suspension mechanism is noted, with information regarding the fitting and manufacturing processes omitted. In this article, the concerns, issues and consequences arising from lack of upper and lower limb socket documentation are discussed from a researcher perspective, supported by healthcare professionals and socket fabrication specialists. Key changes are proposed to the way socket manufacturing and evaluation are documented to assist future research.

## 1. Introduction

The majority of limb prostheses are attached to the human body using a prosthetic socket. For these devices, the socket is one of the most critical components (1–3) for suspension, comfort, and control (1, 4–6).

The socket is unique among prosthetic components, as it is the only part that is not mass-produced, instead it is custom-made for each individual residual limb by a prosthetist or technician (4). The profession of “Prosthetist and Orthotist”, also known as “Orthopaedic Technician”, is legally protected in several nations (7). For countries without protected profession directives, the establishment of international standards and regulatory bodies for devices and training is of utmost importance (8). Although necessary to preserve patient safety, the regulated nature of the profession limits the amount of information that is publicly shared about manufacturing processes. The evidence and description of their workflow is frequently missing from literature, and it is common for research evaluating prosthetic components (e.g., a knee joint) to note the type of socket and suspension mechanism but include no precise information about fit or manufacturing process. Consequently, it is not possible to evaluate to what extent socket fit has impacted the results of studies analyzing other prosthesis-related outcome metrics, such as the dependability of myoelectric control (9).

The socket acts as the interface between the body and prosthesis, facilitating load bearing (1) and proprioception in lower-limb devices. In myoelectric prostheses, the socket houses electrodes which allow the transmission of control signals (10). Despite functional differences between upper and lower limb prostheses, the key factors affecting user acceptance and function are similar (11), with discomfort and diminished prosthesis control being the leading causes of abandonment and dissatisfaction (11, 12).

Each socket's shape is derived from the anatomy, requiring complex design modifications based on factors such as location and level of amputation or limb absence, bony prominences and tissue consistency, the quality and load bearing capabilities of the tissues, and scars (13).

Currently, there is neither a quantifiable nor a universally accepted definition of what constitutes a well-fitting socket. Although guidelines exist regarding how to administer specific socket designs and manage pressure distribution (6, 14), socket creation relies heavily on the skills and knowledge of individual prosthetists gained from years of experience (6, 15, 16), and the subjective feedback from prosthesis users, which makes it difficult to standardize the quality of socket fit (15).

The article aims to highlight the issues caused by the lack of published information surrounding prosthetic socket manufacturing and evaluation—the key stages of prosthetic socket provision identified by the authors. The article takes the format of an opinion piece; a first-hand account from a group of seven biomedical engineering researchers and two clinician researchers (one physiotherapist and one prosthetist). It is hoped that formally documenting some of the key issues will raise questions to inform future research and evidence the requirements to obtain a clear definition of a well-fitting socket. Recommendations are proposed to address the issues discussed in the article to enhance the understanding of socket fit in research.

## 2. Socket Design and Manufacturing

The traditional process for creating a socket is similar for both upper and lower limbs. Prosthetists mark bony prominences and important areas on the limb and then take a Plaster of Paris cast, capturing their markings and hand sculpting the cast as it dries. The negative cast is used to produce a positive plaster replica of the limb which is then rectified (a manual process where material is added and removed to refine the fit and comfort of the socket) (14, 17). The socket, or a temporary diagnostic “check” socket, is then produced and the fit is confirmed by the prosthetist (17–19). However, despite the importance of these checks, many intermediate steps in the socket fitting process are not documented in literature e.g., prosthetists check for pressure and movement within the socket and shaping of the socket trimline, all of which rely heavily on experience (19).

It is plausible that some steps of socket production, such as hand sculpting, cannot be quantified, or even verbalized. The skills required to sculpt and rectify a prosthetic socket go far beyond the initial training given to prosthetists. Achieving a good fit relies directly on tacit knowledge, the prosthetist's experience and the feel of the limb under palpation and manipulation, and its underlying structures.

Digital scanning has become increasingly common as a method of capturing limb geometry, used in conjunction with Computer Aided Design/Manufacturing (CAD/CAM) to create sockets (20, 21). Generally, the aim of digitizing socket manufacture is to minimize workshop time, permit repeated production, provide a more autonomous method of production, and allow rapid prototyping. The process also leaves a perpetual digital record of design. However, to ensure the tactile and tacit experience of clinicians is accurately captured by digital platforms, it should be documented. Until this is done, there will remain a barrier to reliably producing sockets using digital methods such as direct limb scanning and CAD/CAM. Complex digital scanning techniques have been trialed in research (22, 23), and attempts have been made to identify common trends in CAD/CAM socket rectification (24), but a method that incorporates the feel of socket fit is yet to emerge.

As current methods are “tried and tested” there is little information to guide what is “safe” for novel methods and materials. Standard ISO10328:2016 (25) exists for structural testing of lower-limb prosthetic components but does not specifically describe testing of the socket itself, and no such standards exist for upper-limb devices. This limits the ability to screen and verify the safety of new socket designs, materials and fabrication methods like additive manufacturing. Safety is the utmost priority for clinicians; therefore, the lack of published safety guidelines will prevent novel methods such as 3D printing moving from research and private clinics to a mainstream manufacturing method (26).

### 2.1. Workflow Omission in Research Reporting

Prosthetics researchers often rely on the input of a registered prosthetist and in-house prosthetics technicians to manufacture research sockets. The prosthetist/technician's technique is rarely reported in subsequent publications. By omitting their workflow and recording the socket as if it was a prefabricated component, a wealth of knowledge is lost. The decisions that lead to the chosen socket design, including how long the procedure took, the cost, the level of rectification, how many attempts were made to obtain a satisfactory socket fit, and details of each participant's individual limb are rarely, if ever, documented in literature. Omitting the workflow that precedes and covers socket production in research studies may limit their external validity, or the application of their findings.

The mechanical performance of prototype socket designs and materials may differ substantially depending upon the size and shape of the limb for which they are designed. Comparisons about novel materials cannot be made if materials and how they are used (e.g., layers, thickness) are not noted. Additionally, the scarcity of modern workflow documentation from clinics makes room for the misrepresentation of capabilities regarding modern manufacturing methods such as 3D printing and digital scanning. For researchers who work independently from clinicians, this can create the illusion that digital methods are a ready-to-use alternative which does not require clinical intervention. This misconception can be detrimental to clinicians and has the potential to impact patient safety.

### 2.2. Materials

The evolution of materials used to manufacture sockets has been documented for over a century—transitioning from wood, to leather, to modern polymers and carbon fiber (26, 27). However, the reasons for choosing a specific material or the number of layers used in laminates are rarely noted. It is not clear what factors were considered when phasing out older materials or bringing in new ones. Issues of cost, expertise, patient demand, or manufacturing materials are likely to be important but are not noted making transitions or improvements problematic and at times may lead researchers to “re-invent the wheel”.

### 2.3. Evolution of Socket Types

For lower-limb, there have been advancements in the load distributions of sockets (28–33), and suspension mechanisms (2, 34). However, apart from the materials utilized and stand-out novel designs (35), documented upper-limb socket styles and manufacturing techniques have remained relatively unchanged for decades (36). Researchers rely on original design specifications for sockets (28, 29, 37, 38), however, prosthetists will create hybrids and modifications of original designs as they see fit. Such changes have not been documented although are accepted as common knowledge in the field (39).

## 3. Outcome Measurement

To understand the impact of socket design and manufacturing, as well as understand where improvements are needed, it is necessary to evaluate the outcome of the process. This is often undertaken clinically at the individual level by a prosthetist reviewing the fit of the socket and establishing whether the patients finds it comfortable. Currently, socket fit and comfort are assessed using simple scales. However, there is no universally accepted definition for socket comfort or fit. Moreover, there is no single accepted measurement tool for clinicians to assess these outcomes or for users to feedback.

### 3.1. Definition of Socket Fit and Comfort

Often, the terms socket fit and comfort are used interchangeably. Neither term has a universally accepted definition, although it is generally accepted that socket fit refers to diverse metrics assessed by a prosthetist regarding the volume and shape match to the limb, safety and suspension, whereas socket comfort is a subjective measure reported by the prosthesis user.

There are no defined rules for what constitutes a good socket fit, hence there is sparse literature detailing how to assess whether a socket is well-fitting. Interface stress (the pressure and shear between the socket and residual limb) has been identified as an important factor (3, 11, 40). Whilst little is defined about good fit, much is known about the context of poor fit. Most literature refers to the medical consequences of ill-fitting sockets or inadequate distribution of load, e.g., skin breakdown and deep tissue injury (41–44). Little is documented about the day-to-day impact on quality-of-life stemming from poor socket fit (3, 45).

Socket comfort consistently ranks highly as a factor contributing to prosthesis abandonment (12, 46). Socket comfort can be used as a measure of socket fit (47), with correlation between user-reported comfort scores and prosthetist evaluation of the socket fit. However, first-hand experience working with people with lower-limb amputations who self-report high levels of comfort and satisfaction, suggests that this is not always the case, but no documented examples have been found.

### 3.2. Socket Satisfaction Measurement

Subjective metrics such as comfort are important in the socket fitting process, since user approval is crucial for prosthesis acceptance. Without user acceptance of the socket, a prosthesis is likely to be abandoned regardless of other metrics. Attempts have been made to quantify socket comfort in the form of standardized scales in evaluation questionnaires. To date, the Socket Comfort Score (SCS), and Comprehensive Lower-Limb Amputee Socket Survey (CLASS) are the only scales which specifically address socket comfort (47, 48). The SCS and CLASS do not report the cause of discomfort, simply the presence and magnitude, and are validated for use with lower-limb prosthesis users only. Metrics such as the Trinity Amputation and Prosthesis Experience Scales (TAPES) (49) and Prosthesis Evaluation Questionnaire (PEQ) (50) have high validity and ask specific questions about socket fit. However, they do not provide an in-depth assessment of socket comfort, instead focusing on overall wellbeing and general satisfaction with the prosthesis. There is a lack of consensus around what aspects should be measured and how (51), evidenced by the different focus of existing tools.

Currently, a disconnect exists between the methods used in research and those used in clinical practice to evaluate socket satisfaction at the point of provision. Research focus tends to revolve around assessing and correcting interface pressures with novel technology, based on objective outcome measures (52–54). However, in clinics, assessment of interface pressures is generally done without specialist equipment; instead, subjective measures such as monitoring the redness of a residual limb after doffing the prosthesis are commonly used in combination with patient feedback (35). Measurement scales may be used to evaluate socket comfort; however, this can be misleading due to varying personal experiences with pain (55), as well as individuals who have reduced sensation in their residual limb (56). For this reason, the changes in scores are often considered more pertinent to consider than the absolute scores reported.

Communication between patients and their clinicians is important in the evaluation of socket comfort. The importance of language to communicate subjective experiences has been illustrated by Bourke in the context of pain (55). The words chosen by patients to communicate the same experience may differ to each other. For example, to one person a given level of pressure may be discomfort, whereas to another it may be pain. The interpretation of the clinician is also a factor in understanding the experiences of the patient.

There are currently no scales in existence which allow qualitative measures, such as user explanations for their responses or clinician notes to be taken into context, which has led some researchers to develop bespoke surveys to document socket fit, comfort and its primary impacts (3, 17). In recent years, research groups have called for the introduction of improved quantifiable outcome measures of patient socket satisfaction (57, 58). In terms of gauging socket satisfaction, none of the existing scales are internationally recognized as the “standard” metric, hence it is difficult to draw comparisons between the outcomes of various novel prosthetic interventions. Documentation of the usage of SCS and similar scales within clinics has not yet been reported, making it difficult to assess whether any existing scales should be adopted as a universal metric. Moreover, there are no scales designed specifically to assess comfort for upper-limb sockets.

### 3.3. Outcome Sharing

The current evaluation measures for socket satisfaction only gauge how the wearer is feeling at the time of the assessment, but do not give a detailed picture of the changes that may occur as the person uses their limb in their daily activities. Without data on how socket satisfaction changes over time and the impacts this has on the wearer's day-to-day activities, a wealth of knowledge and the potential for learning from past interventions is lost. In addition to this, the reasons for which sockets are revised (e.g., limb volume changes, mechanical failure, discomfort) are not widely documented.

In order to assess novel socket designs, a comparison of their performance against a similar, existing socket is required. Current comparisons rely on case-studies, as large amounts of data relating to the performance of specific socket types is not accessible. Atypical and complex socket fitting cases are common, and certain levels of amputation are much less prevalent, e.g., upper-limb amputation (26, 59–61). Therefore, it would be beneficial to have global pooled data sharing between clinics and researchers to facilitate a greater understanding of sockets prescribed and trialed for different circumstances. However, for the sharing of clinical outcomes to be possible, it is necessary to have a standardized protocol for what information should be collected and how, and which outcome measurement tool should be used.

## 4. Proposed Recommendations

The issues raised in this article are key inhibitors for the development of new socket technology, and the interpretation of wider prosthetics research. The common theme is how sockets are documented, irrespective of the stage at which they occur in the limb provision process.

At the socket design and manufacturing stage, up-to-date technical specifications of socket designs, alongside an overview of modern manufacturing and decision-making processes should be made accessible. To enable this, a set of standardized reporting guidelines for evaluating novel prosthetic elements should be produced. In addition, the establishment of an ISO standard for structural testing of sockets and upper-limb prostheses equivalent to that of ISO 10328:2016 would also be useful.

To allow clear communication in prosthetics research and practice, a greater understanding of socket fit and socket comfort, from clinician and user perspectives, is needed in order to establish agreed definitions. This would enable a standardized clinical assessment of both socket fit and comfort. The scales should be validated for both upper and lower limb sockets, accounting for their different functional requirements.

Reporting guidelines should be created to facilitate global data sharing of socket fitting case studies, alongside the reported success of each fitting. For this to be possible, a standardized approach for outcome assessment should be established, including whether the socket provision was successful (and what success means), reported feedback from the user and what special measures were taken to enhance the fit. Sharing of clinical outcome assessments would be beneficial to research articles exploring the outcome of prosthetic socket manufacture, design, and fitting, but also the evaluation of other prosthetic components. This sharing of information could influence best practice and lead to improvements in both clinical practice and research. A standardized protocol would allow research to be comparable and heighten the value of evidence.

## 5. Conclusions

This article proposes there should be detailed published information regarding the elements of socket design and fabrication that are currently based upon implicit knowledge. It provides a call for a clear, universal definition of what constitutes a good socket fit and how it differs from comfort, based on an in depth understanding of clinician and user experience. Finally, it highlights the need for universally accepted outcome measures to evaluate the fit of a prosthetic socket and enhanced data sharing between clinics and researchers.

## Data Availability Statement

The original contributions presented in the study are included in the article/supplementary material, further inquiries can be directed to the corresponding author/s.

## Author Contributions

JO and ST: conceptualization and writing—original draft preparation and editing. AC, AD, CO, LA, AM, SDu, and SDa: writing—review and editing. All authors have read and agreed to the published version of the manuscript.

## Funding

This work was supported by the Engineering and Physical Sciences Research Council (EPSRC), U.K., under studentship number 2281137 from EP/N509528/1 and EP/R51309X/1 (JO), EP/R014213/1 (AD and CO), and Scar Free Foundation, no grant number given (ST and AM).

## Conflict of Interest

The authors declare that the research was conducted in the absence of any commercial or financial relationships that could be construed as a potential conflict of interest.

## Publisher's Note

All claims expressed in this article are solely those of the authors and do not necessarily represent those of their affiliated organizations, or those of the publisher, the editors and the reviewers. Any product that may be evaluated in this article, or claim that may be made by its manufacturer, is not guaranteed or endorsed by the publisher.
